# Correlation of proteome-wide changes with social immunity behaviors provides insight into resistance to the parasitic mite, *Varroa destructor*, in the honey bee (*Apis mellifera*)

**DOI:** 10.1186/gb-2012-13-9-r81

**Published:** 2012-09-28

**Authors:** Robert Parker, M Marta Guarna, Andony P Melathopoulos, Kyung-Mee Moon, Rick White, Elizabeth Huxter, Stephen F Pernal, Leonard J Foster

**Affiliations:** 1 University of British Columbia, Centre for High-Throughput Biology and Department of Biochemistry & Molecular Biology, 2125 East Mall, Vancouver, BC, V6T 14, Canada; 2 Agriculture & Agri-Food Canada, Beaverlodge Research Farm, PO Box 29, Beaverlodge, AB, T0H 0C0, Canada; 3 University of British Columbia, Department of Statistics, 2207 Main Mall, Vancouver, BC, V6T 1Z4, Canada; 4 Kettle Valley Queens, 4880 Well Rd., Grand Forks, BC, V0H 1H5, Canada

**Keywords:** Honey bee, Proteomics, Social immunity, Hygienic behavior, *Varroa *sensitive hygiene

## Abstract

**Background:**

Disease is a major factor driving the evolution of many organisms. In honey bees, selection for social behavioral responses is the primary adaptive process facilitating disease resistance. One such process, hygienic behavior, enables bees to resist multiple diseases, including the damaging parasitic mite *Varroa destructor*. The genetic elements and biochemical factors that drive the expression of these adaptations are currently unknown. Proteomics provides a tool to identify proteins that control behavioral processes, and these proteins can be used as biomarkers to aid identification of disease tolerant colonies.

**Results:**

We sampled a large cohort of commercial queen lineages, recording overall mite infestation, hygiene, and the specific hygienic response to *V. destructor*. We performed proteome-wide correlation analyses in larval integument and adult antennae, identifying several proteins highly predictive of behavior and reduced hive infestation. In the larva, response to wounding was identified as a key adaptive process leading to reduced infestation, and chitin biosynthesis and immune responses appear to represent important disease resistant adaptations. The speed of hygienic behavior may be underpinned by changes in the antenna proteome, and chemosensory and neurological processes could also provide specificity for detection of *V. destructor *in antennae.

**Conclusions:**

Our results provide, for the first time, some insight into how complex behavioural adaptations manifest in the proteome of honey bees. The most important biochemical correlations provide clues as to the underlying molecular mechanisms of social and innate immunity of honey bees. Such changes are indicative of potential divergence in processes controlling the hive-worker maturation.

## Background

Social insects such as the honey bee (*Apis mellifera *L.) derive great benefit from living in tight-knit groups that enable greater efficiencies in brood care, foraging and defense against predation. However, the high population densities and relatedness of individuals leave colonies susceptible to emerging infectious diseases [1]. *Varroa destructo*r, an ectoparasitic mite of the honey bee [2] causes varroasis, which is a leading contributor to ongoing colony losses in commercial apiculture worldwide [3]. *V. destructor *feeds on the hemolymph of larval and adult bees, inflicting nutritional stress and immune suppression, as well as acting as a major vector for viral pathogen transmission [4].

In solitary insects, cellular or humoral-based defenses provide the only known system for immunity, but *A. mellifera*'s genome reveals that while honey bees contain these systems for immunity, the number of immunity genes is lower than that of solitary insects such as flies, moths and mosquitoes [5]. As an apparent compensation for this, social insects have evolved collective systems of behavior that provide defenses against disease and parasitism. Two related behaviors, hygienic behavior (HB) and *Varroa *sensitive hygiene (VSH), are highly variable among *A. mellifera *colonies and are seen as important traits in the development of disease and mite-resistant stock. HB is a well-documented protective behavior that involves nurse-aged worker bees uncapping brood cells and removing parasitized or diseased pupae [6]. VSH is less well-understood but it encompasses a suite of behaviors that ultimately suppress mite reproduction by uncapping and/or removing mite-infested pupae from sealed brood resulting in a high proportion of non-reproductive mites in the brood that remains [7,8]. HB and VSH can be quantified using field assays and are heritable so, while both are now used in the selective breeding of *Varroa*-resistant bees [9,10], the genetic and biochemical mechanisms that drive them are poorly resolved.

To date, most selective breeding in commercial apiculture focuses on traits such as honey production, color, gentleness, winter survival or other economic parameters. When combined with continual dilution of the gene pool through importation of susceptible stock, these selections limit host adaptation to pathogens. In order to improve disease and mite tolerance, field assays for HB and VSH must be incorporated into the stock selection process [11,12]; however, these assays are resource intensive, lack sensitivity and may require closed breeding [13], limiting their suitability for widespread application. To support the creation of novel assays, a molecular-level mechanistic understanding of resistance traits is seen as a promising avenue to support commercial breeding and disease prevention through marker-assisted selection (MAS) [14]. To date, low-resolution microsatellite-based quantitative trait loci (QTL) for HB have been reported [15], as have some of the biochemical consequences to the host of infection by *V. destructor *and associated viruses [16,17]. Transcriptome changes in *A. mellifera *and in *Varroa's *natural host *A. cerana *also pinpoint subtle changes in transcript expression for components responsible for neuronal rewiring, olfaction, metabolism and aspects of social behavior that may be critical components driving mechanisms of *Varroa *tolerance [18,19].

All the molecular investigations of HB and VSH have used well-controlled colonies or individual bees without examining the natural variation and distribution of both the traits and their molecular components. Thus, here we tested the hypothesis that inter-colony variation in disease resistance parameters is reflected by changes in the expression of specific proteins. Sampling from a large cohort of colonies, we measured the relative abundance of approximately 1,200 proteins from two bee tissues involved in interactions with the pathogens and correlated these with estimates for active bee behavioral phenotypes for HB and VSH, as well as host-pathogen population dynamics. Through meta-analysis of these data with other available information, proteins and biochemical processes most likely to be responsible for the observed disease resistance traits were identified.

## Results

### Behavioral phenotypes and mite-bee dynamics

**Figure 1:**
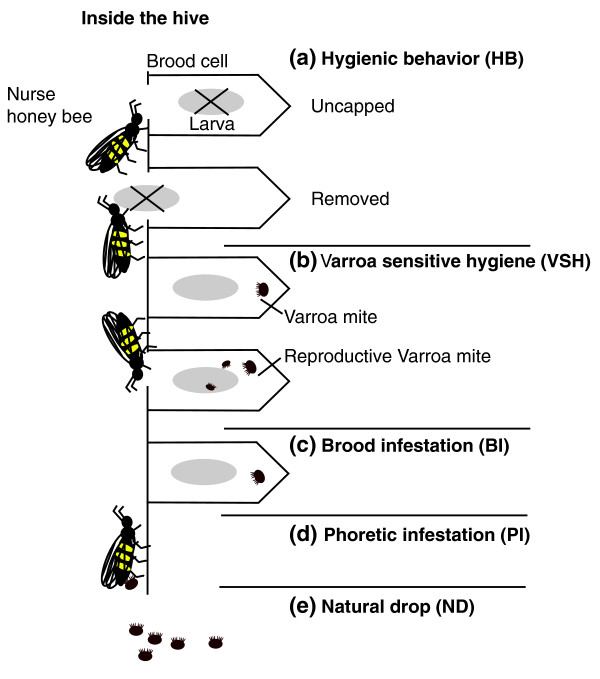
**Diagram depicting honey bee disease tolerant traits and infestation dynamics**. (**a) **Hygienic behavior (HB) is composed of two component behaviors, 'uncapping' (uncapped, U) which involves the opening of the cell containing a dead pupa and 'removal' (removed, R) which involves the removal of the dead pupa from the cell after uncapping has occurred. These behaviors are not always performed by the same bee. HB was recorded over 24 hour (rapid) and 48 hour (slow) periods. (**b**) Varroa sensitive hygiene (VSH) was defined by determining the proportion of *Varroa*-infested cells in which no reproductively viable *Varroa *mite daughters were produced. Increases in this measure infer that greater proportions of mites remaining in the brood have had their reproduction suppressed because of infertility, death, the production of only males, or have had their reproduction delayed preventing sexual maturation of females. **(c) **Brood infestation (BI) is the percentage of brood cells infested by one or more mites regardless of the mite's reproductive status. **(d) **Phoretic infestation (PI) is an estimate of the density of mite phoresy on adult bees, and **(e) **natural drop (ND) is a normalized measure of the number of mites falling from the adult bees onto an adhesive board on the bottom board of colonies.

**Figure 2:**
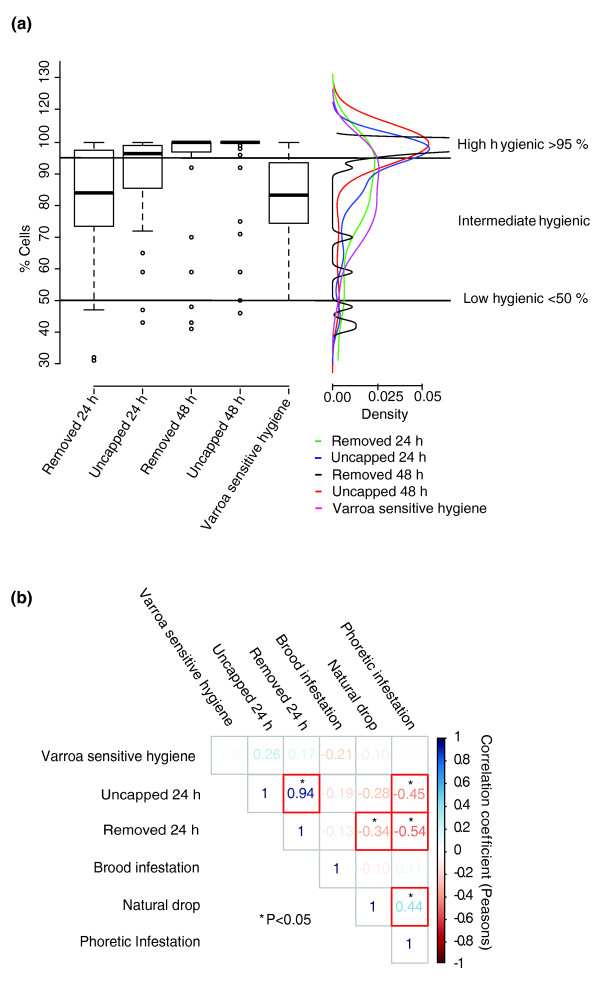
**Distribution of and correlation between behavioral traits**. **(a) **Box and whisker, and density plot for disease tolerant traits for hygiene at different times after the introduction of freeze killed brood and the measure for VSH. **(b) **Correlation matrix showing the correlation score and significance for estimates of rapid hygienic behavior and VSH with measures of infestation. VSH, Varros sensitive hygiene.

To assess the expression of disease tolerant behaviors, colony-level measurements of various metrics of HB, VSH and mite-host population dynamics were made (Figure 1), including phoretic infestation (PH, mites on adult bees), natural mite drop (ND, estimate of mite death rate), and levels of brood infestation (BI). HB was estimated by observing the proportion of a defined number of freeze-killed, sealed brood cells that bees first uncapped (uncapped, U) and then removed (removed, R) at 24 and 48 hours (Figure 2a). The hygienic response to freeze-killed brood was time dependent, with widely distributed levels of HB at 24 hours and the majority of hives achieving the accepted 95% threshold for the proportion of removed cells at 48 hours [11]. Because colony scores in the other measured parameters were distributed roughly similarly to HB at 24 hours as indicated in the kernel density plot of Figure 2b, we asked which of them were independent measures and which were interrelated. Pearson's product-moment correlation (PPMC) coefficient revealed a statistically significant negative pair-wise dependence between estimates for hygiene and mite infestation dynamics (PH, ND) (Figure 2b). Maximum dependence was observed between cell removal and PH (r = -0.54, *P *= 0.0007), confirming that colonies better able to detect and uncap cells with affected brood are able to reduce adult infestations more efficiently. Observing differences in density for temporal aspects of hygiene and interactions between disease-tolerant behaviors and infestation indicated we were detecting natural variation in the speed of HB removal that directly influenced the colonies' tolerance to *Varroa *mites.

### Correlation of protein expression and behavior

While most of the parameters discussed above are known to have a genetic basis, all must ultimately manifest as the result of changes in protein expression and/or activity. To explore potential mechanisms underpinning natural variation in *Varroa *tolerance across these colonies, we examined the protein expression profiles of two tissues that play a critical role in the bee-*Varroa *interaction: antennae of brood-nest workers (that is, mostly nurse bees) and the integument from fifth instar worker larvae. Antennae were used because they are adult bees' primary sensory organs and many of the behaviors evaluated here involve bees being able to sense the presence of either the pathogen itself or a damaged/diseased nest-mate. Integument was chosen because it is the initial physical barrier to *Varroa *when they feed on larvae and as such the innate processes found here may be critical components in the response of hygienic adults and provide direct innate mechanisms of tolerance. It is possible that changes in the composition of larval proteins or the metabolites produced by these proteins during infection may trigger HB or VSH in adult nurses.

**Figure 3:**
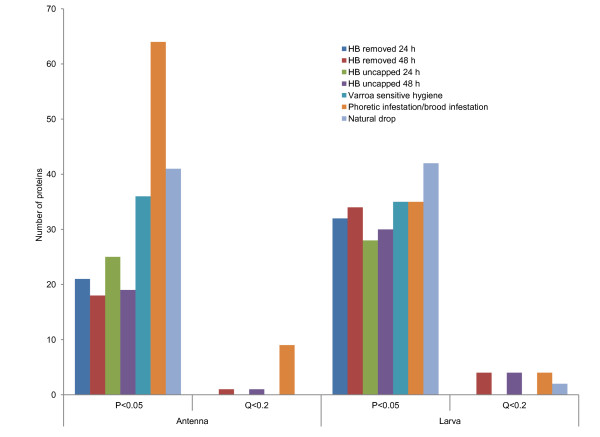
**Results of proteome correlation screen for antennal and larval tissues**. Bar plot giving the number of proteins found correlating with traits at different cut-off's for statistical confidence.

Using liquid chromatography-tandem mass spectrometry (LC-MS/MS) to analyze three independent samples of each tissue, we constructed protein expression profiles for approximately 1,200 proteins across all colonies as described previously [20]. By centering and standardizing across labels and colonies, the relative expression ratios from individual LC-MS/MS experiments are converted into a roughly normalized distribution of protein effect, representing the expression level of each protein in each colony relative to the population average. These variables (response variable) were then regressed against the behavior and infestation estimates (predictor variable) measured for that colony. The direction of each regression was determined by the sign of the estimated regression coefficient and the significance of that effect was accessed using a mixed linear model with probability cut-off at Q <.2 adjusting for multiple comparisons or later *P *< .05 for explorative data analysis (see Figure 3).

### Several proteins are highly significant predictors of resistance to Varroa mite infestation

**Figure 4:**
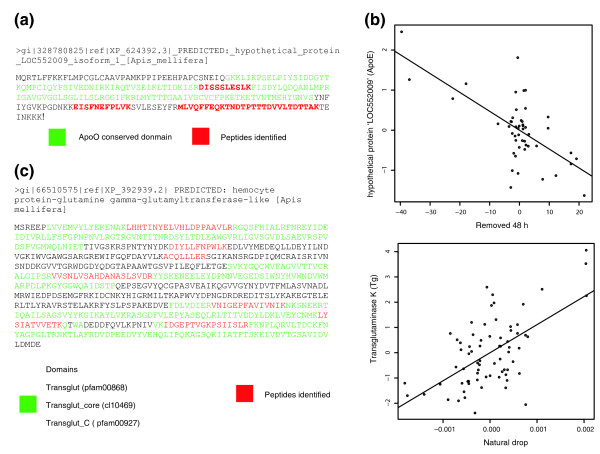
**Protein sequence, peptide identification coverage, and conserved domains for two proteins with highly significant correlation with *Varroa *tolerant traits**. **(a-b) **Sequence encodes a probable ApoO type protein found to correlate with hygienic behavior at 48 hours. **(c-d) **A transglutaminase (Tg) correlates with natural drops, an estimate of mite death rate in the colony. ApoO, apolipoprotein O.

To adjust significance levels to account for the multiple-testing hypothesis, proteins were filtered using Q <.2 cut-off; for HB one antennal protein and five larval proteins survived this additional filter (Tables 1, 2). In the antennae, the hypothetical protein 'LOC552009' of unknown function correlated with HB at 48 hours (Q = 0.09) for both 'uncapped' and 'removed' behaviors. Sequence analysis revealed that LOC552009 contains a conserved domain similar to the mammalian protein lipid transport protein Apolipoprotein O (ApoO) [21]. Figure 4a, b shows the added variable plot for this protein correlating with HB (removed 48 hours), peptides identified and protein sequence containing the conserved domain for ApoE.

**Table 1:** Name, expression and function of larval proteins found correlating with traits measured for resistance to *Varroa *infestation.

Protein Name	R48	U48	PH/BI	ND	Function
similar to CG5903-PA	↓	↓	-	-	CDD:ApoE (Apolipoprotein, lipid transport)
PREDICTED: polyadenylate-binding protein 1-like isoform 1	-	-	↓	-	mRNA surveillance pathway
PREDICTED: 60S ribosomal protein L14	-	-	↓	-	Translation
PREDICTED: alpha-tocopherol transfer protein-like			↓		Vitamin E distribution
PREDICTED: alcohol dehydrogenase [NADP+] A-like			↓		Alcohol metabolism
phenoloxidase subunit A3			↑		Monooxygenase, phenoloxidase activity
PREDICTED: dehydrogenase/reductase SDR family member 11-like	-	-	↑	-	Oxidoreductase activity (Metabolism)
PREDICTED: v-type proton ATPase subunit F 1-like			↑		Oxidative phosphorylation (Electrom transport)
PREDICTED: calcyphosin-like protein-like	-	-	↑	-	Intracellular signal transduction
PREDICTED: sorbitol dehydrogenase-like isoform 2	-	-	↑	-	Fructose and mannose metabolism

BI, brood infestation; ND, natural drop, PH phoretic infestation; R, removed; U, uncapped.

**Table 2:** Name, expression and function of antennal proteins found correlating with traits measured for resistance to *Varroa *infestation.

Protein name	R48	U48	PH/BI	ND	Function
eukaryotic translation initiation factor 3 subunit A	↓	↓	-	-	Mitotic spindle elongation; mitotic spindle organization
rab proteins geranylgeranyltransferase component A 1	↓	↓	-	-	Sensory transduction, intracellular transport
argininosuccinate synthase-like		↓	-	-	Alanine, aspartate and glutamate metabolism
glucose-6-phosphate 1-epimerase-like	↑	↑	-	-	Glycolysis/Gluconeogenesis
UDP-glucose:glycoprotein glucosyltransferase	↑	-	-	-	Protein glycosylation
mitochondrial import inner membrane translocase subunit Tim8	-	-	↓	-	Protein import into mitochondrial inner membrane
60S ribosomal protein L10 isoform 1	-	-	↓	-	n/a
26S proteasome non-ATPase regulatory subunit 2	-	-	↑	-	Regulatory subunit of the 26 proteasome
hemocyte protein-glutamine gamma-glutamyltransferase	-	-	↑	↑	Peptide cross-linking, emolymph coagulation
					

BI, brood infestation; ND, natural drop, PH phoretic infestation; R, removed; U, uncapped.

In larvae, several more candidate proteins were identified as strong positive and negative predictors for HB (Table 2), suggesting that events in the larvae may be able to influence HB of the adult. Further correlation analysis of mite infestation/fertility measures (PH, BI, ND and VSH) identified the hemocyte protein-glutamine gamma-glutamyltransferase-like (a putative transglutaminase) as highly significant (Q = 0.02) and positively correlated with ND (Figure 4c).

To increase the specificity of our measures for infestation dynamics, we next calculated the ratio of mites observed phoretically to those found in brood cells (PH/BI). This adjustment enabled quantitation of the relationship between two important stages in the mite life cycle, where long phoretic phases may be indicative of poor reproductive success and the influence of adult bee behavior or larval attractiveness. After adjustment, several proteins (nine in antenna, four in larva) were highly significant (Q <.2) in both tissues. Importantly, the adjusted metric was also highly correlated with ND (Pearsons product correlation: t = 5.2211, df = 36, P = 7.633e-06) and in larva correlated with increased significance with the protein Tg (Q = 0.01), supporting the role of Tg as an accurate measure of *Varroa *resistance (Figure 4, Tables 1, 2). Tg also provides the clearest link to phenotype. In insects, Tg is the primary protein facilitating the crosslinking of the clotting factors, hemolectin and Eig71Ee. Tg drives clot formation, an important defensive process against ectoparasites such as the *Varroa *mite. Of the proteins correlating in the antenna, several form part of diverse metabolic pathways and are impossible to link to function at this point. However, calcyphosin-like protein and phenoloxidase subunit A3 are worthy of discussion. Calcyphosin (Q = 0.02) has been found expressed in olfactory cells in lobster and with a putative role in signal transduction in sensory cells. Phenoloxidases (Q = 0.1) are a critical part of insect immunity during the pathogen encapsulation response, but are also important in the ecdysteroid-dependent processes linked to polyphenism and caste differentiation.

### Neuronal proteins underpin hygienic behavior and VSH in antennae

The proteins discussed above that survive correction for multiple hypothesis testing should be excellent predictors of HB, perhaps even usable in marker-assisted selective breeding. To find so many highly significant proteins in a completely out-bred population is remarkable but the requirements that they must pass mean that it may be too restrictive a dataset to understand fully some of the molecular mechanisms underlying the relevant behaviors. To this end, we expanded the analysis to discover processes with mechanistic relevance for HB and VSH. Those proteins with a significant correlation (*P *< .05) to one or more behavioral traits were explored using ontological classifications provided by the honey bee refseq entry in the National Center for Biotechnology Information (NCBI) or the flybase homolog. The most significant of these enrichments (*P *< .05, Table 3) largely tracked with the altered distributions for estimates of HB, suggesting some of the molecular mechanisms that may regulate this behavior. The set of proteins highly correlated with rapid HB (>95% removed by 24 hours) was particularly enriched for proteins involved in 'sensory development'. At 24 hours, both uncapping and removal traits correlated with the up-regulation of the secretory proteins windbeutel, amphiphysin (Amph) and [RefSeq:CG6259] which encodes the homolog to human CHMP5 protein. Proteins down regulated in rapid hygienic bees were ankyrin 2, laminin A, Zasp (Z band alternatively spliced PDZ-motif protein) and fasciclin 1 (Fas1) all involved in 'cell adhesion'. Proteins correlating with both elements of HB at 48 hours (>95% open/cleaned 48 h ours) were enriched in the ontology 'mitochondrial inner membrane'. This ontology was also identified for the rapid uncapping behavior and corresponds to reduction in primary metabolic pathways.

**Table 3:** Summary of gene enrichments for larval proteins correlating with anti-parasitic traits.

Behavior	Expression	Enriched ontologies
**R24**	↑7	Macromolecular localization (wbl, Amph, Hel25E, CG6259), protein localization, sensory organ development (wbl, Amph)
	↓13	tissue development, nerve growth factors (Ank2, LanA), axon guidance, cell adhesion (Ank2, LanA, Fas1, Zasp52, RpS25)
**U24**	↑11	Macromolecular localization, protein transport (wbl, Amph, Hel25E, CG6259), microtubule cytoskeletal organisation, cell cycle (RpS30, RpL12, Hel25E, Rab11)
	↓14	Cellular respiration (Gdh, ND42 CG5703 Cg6463), mitochodrial electron transport (Gdh, ND42 CG5703)
**R48**	↑9	Translation, mitotic spindle organization (Aats-cys, RpL21, Cctgamma)
	↓9	Mitochondrial inner membrane (sesB, ND42, CG5703, I(2)06225)
**U48**	↑12	No enrichment
	↓7	Mitochondrial inner membrane (ND42, CG5703)
**VSH**	↑14	Ubiquitin-dependent protein catabolic process (Rad23, Prosalpha7)
	↓20	Cytokinesis (Gammacop, SNAP)

R, removed; U, uncapped; VSH, Varroa sensitive hygiene.

**Figure 5:**
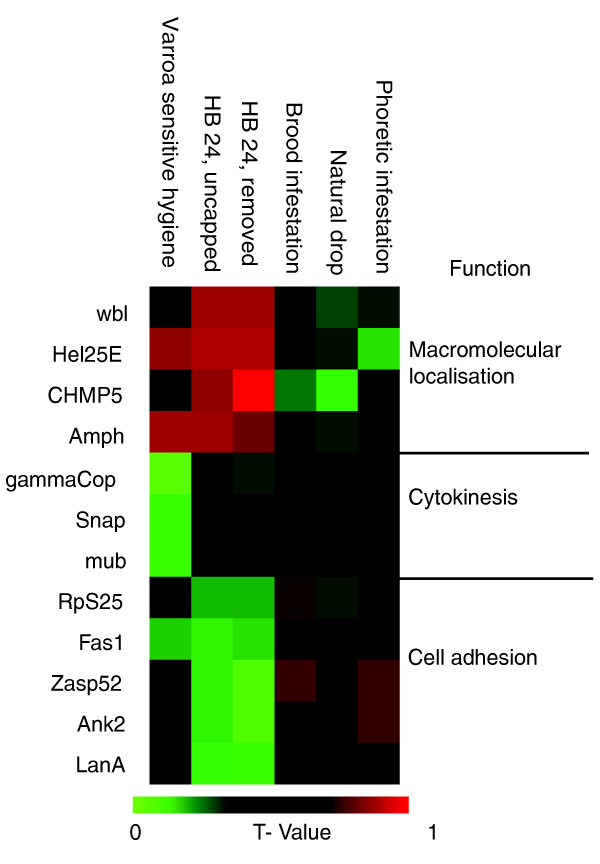
**Heat map of t values for proteins with involvement in neuronal/synaptic function in antennae found to correlate significantly with hygienic behavior and VSH**. VSH, Varroa sensitive hygiene.

VSH and HB correlated strongly with some of the same proteins, even though there was no apparent interdependence between VSH and HB (Figure 5). Proteins that correlated with VSH and HB included Fas1, which was negatively correlated with rapid HB and VSH, while Amph and helicase 25E (Hel25E) levels were significant positive predictors of both VSH and rapid HB (Figure 5). In addition, several unique proteins with roles in synaptic function correlated only with VSH. VSH was negatively correlated with expression of the mushroom body protein (Mub), soluble NSF attachment protein (SNAP) and gammaCOP. Mub is involved in temperature preference in *Drosophila*, [22] while SNAP is a key pre-synaptic protein mediating synaptic vesicle fusion and gammaCOP is involved in vesicle trafficking at synapses and other vesicle sorting pathways [23]. The protein with greatest change in expression with respect to VSH measurements was [RefSeq:LOC412768], a poorly annotated member of the take-out/juvenile hormone binding protein (To/JHBP) superfamily involved in chemoreception.

### Larval proteins with cuticular and immune function correlate with HB and VSH

HB and VSH are considered to be specific behavioral adaptations of the adult honey bee to diseased brood. However, as our molecular understanding of these traits improves, it is possible that factors expressed within the larva in response to the pathogens may influence the manifestation of the relevant behaviors. Protein/behavior correlations of larval proteins revealed enrichment of chitin-based cuticle structural proteins, particularly cuticular proteins 3 and 13 (CRP3 and CRP13; Table 4). Larval expression of the peptidyl-amino acid modifying enzymes Caf1 and [RefSeq:CG6370] was also positively correlated with HB. CG6370 encodes a dolichyl-diphosphooligosaccharide-protein glycosyltransferase involved in N-glycan biosynthesis in the lumen of the endoplasmic reticulum and, interestingly, four other lumenal proteins were also positively correlated. The larval protein/behavior correlation with the steepest slope was [RefSeq:CG1318-PA], a beta-N-acetyl-D-hexosaminidase with broad substrate specificity ranging from *N*-glycans to chitooligosaccharides. Three proteins clustered under the term carbohydrate metabolism were negatively correlated with VSH in larval tissue. Ecdysone-inducible gene (ImpL3), phosphoglucomutase (Pgm) and gene analogous to small peritrophins (Gasp) all process carbohydrates. Gasps specifically bind or regulate chitin structure in developing embryos, ImpL3 is a lactate dehydrogenase induced by the prohormone of 20-hydroxyecdysone that regulates insect molting, while Pgm is a glycolytic enzyme, a process vital for the biosynthesis of chitin from glycogen. Taken together, these observations suggest a role for larval chitin biosynthesis and/or structural regulation in hygienic and VSH behavior. In the proteins positively correlated with VSH, gene enrichment identified casein kinase II, alpha 1 polypeptide isoform 1 (CkIIapha1 or CK2) and peptidoglycan-recognition protein-SA (PGRP-SA). Both are components of the 'cell surface receptor linked signaling pathway' ontology and effectors of interferon and lipopolysaccharide (LPS) macrophage inflammatory signaling [24]. PGRP-SA detects Lys-type peptidoglycan (PG) from gram-positive bacteria, leading to activation of the Toll receptor pathway and, ultimately, to increased expression of antimicrobial peptides [25,26]. The larval inflammatory response may serve not only as an individual defense mechanism but also as an initiator of social immunity behavior, that is, VSH.

**Table 4:** Summary of gene enrichments for antennal proteins correlating with anti-parasitic traits.

Behavior	Expression	Larval Tissue Enriched Gene ontologies
**R24**	↑18	Peptidyl-amino acid modification (caf1, Cg6370), structural constituent of chitin-based cuticle (Cpr65Av, Ccp84Ae), membrane enclosed lumen (CG1140, CG2118, Adk Caf1)
	↓16	Protein modification process (Rep, Art, Pp2A-29B), regulation of neuro transmitter release (Rep, beta-spec), phosphorous metabolic process (Pp2A-29B, CG7712, PyK),
**U24**	↑18	Peptidyl-amino acid modification (caf1, Cg6370), cell division (Caf1, hts), cell periphery (Hexo1, NepYr)
	↓10	Protein modification process (Rep, Ugt, eff), regulation of neurotransmitter release (Rep, beta-spec), nervous system development (beta-spec, eff, RpL30)
**R48**	↑12	Regulation of embryonic development (Hrb27C, Cg18811), oogenesis (hts, Hrb27C, nudC)
	↓18	Muscle tissue development (beta-Spec, alt, wupA), neurological system process (Rep, alt best-spec), cytoskeletal organization (Beta-spec, alt, cher, Rpl32, Rpl30, eIF3-S10)
**U48**	↑13	Regulation of embryonic development (Hrb27C, CG18811), oogenesis (nudC, hts, Hrb27C)
	↓17	Muscle tissue development (alt, wupA), ligase activity, forming carbon-nitrogen bonds (pug, CG1315)
**VSH**	↑18	Cell surface receptor linked signaling pathway (PGRP-SA, CkIIapla), bristle development (ChIIalpha, CG12163) Translation elongation (Ef1beta, Ef2b)
	↓15	carbohydrate metabolic process (ImpL3, Pgm, Gasp)

R, removed; U, uncapped; VSH, Varroa sensitive hygiene.

## Discussion

We have described here the discovery of several proteins whose expression levels may impact honey bee resistance to infestation by the *Varroa *mite. Natural diversity in these behaviors was a prerequisite to this study and we observed that the levels of each behavior in any given colony were not random. As expected, there was a strong negative correlation between mite infestation levels and HB. At the expression level, several proteins were highly significant predictors of HB and mite infestation dynamics. Highlighted within these proteins were the putative ApoO homolog and a putative Tg. Apolipoproteins are called apolipophorins in insects, and they have diverse roles in lipid solubilization and the transport of small hydrophobic ligands [27-29]. In innate immunity the apolipophorin ApoLp-III stimulates antimicrobial activity in the hemolymph, acting as a pattern recognition system for LPS and lipoteichoic acid (LTA) [29]. Lastly, the strong correlation of Tg with both NDs and an increase in the ratio of phoretic mites to brood mites suggests that Tg activity could provide a measure of resistance to *Varroa *reproduction.

*V. destructor *is an ecto-parasite feeding communally and repeatedly on hemolymph of the honey bee through a bite wound in the cuticle [30-32]. In insects' innate immunity the cuticle provides the first line of defense; once breeched, innate defense systems of the haemocoel cavity are orchestrated by hemocytes, the fat body and hemocoel [33]. Normal wounds heal as hemocytes and plasmatocytes exocytose the clotting factors hemolectin and Eig71Ee [34]. These molecules and other plasma-based factors such as fondue are cross-linked by Tgs in a Ca^2+ ^dependent mechanism to form a primary clot. However, *V. destructor *transmits bio-active compounds that prevent healing and allow continued feeding to occur at the same wound [35]. In the tick arthropod-mammalian ecto-parasitic systems, 18 known bio-active suppressants target innate antiseptic defenses, including several immune cells types, inflammatory and coagulatory cascades [36]. In honeybees, the effect *V. destructor *elicits on the immune system is uncertain. Yang and Cox-Foster [37] demonstrated that *Varroa *parasitism increases the susceptibility of adult bees to bacterial infection, but no major immunosuppressive effects were revealed by transcriptomic studies on specific immune genes or in global analyses [38,39]. More recently a study has reveled that salivary secretions from the *Varroa *mite are able to damage hemocyte aggregation in the tomato moth, (*Lacanobia oleracea*) [38] but no known factors of either pathogen or host are identified. We report here that elevated expression of a putative key clotting factor (Tg) is found in the larva of *Varroa *resistant bee colonies. These data indicate that honey bees have adapted to *Varroa*, increasing the clotting capacity of hemolymph in order to limit mite reproduction.

While the experiments described here were clearly of sufficient power to permit the discovery of some correlations between protein expression and behavioral traits, the variability within such out-bred populations is very high. This is likely a significant limitation in fully defining the molecular mechanism of something as complex as a behavior. Practical limitations in the number of colonies that could be sampled and the depth to which the proteome could be measured across multiple samples were inherent problems here, as with any proteomics study. Even so, an exploratory approach was seen as an important step in generating new hypotheses in a currently poorly understood area of biology.

It is thought that the speed with which hygienic bees respond is driven by a lower limit of olfactory detection of the diseased brood odor [40], which is in turn influenced by the neuromodulator octopamine [41]. In the antennal lobe, octopamine concentration varies between behavioral state, being low in nurse bees and high in foragers. Juvenile hormone and brood pheromone both modulate behavioral responses to octopamine [42] and both are involved in several aspects of behavioral maturation, with the best-understood system being the transition from nurse to forager. This maturation invokes physiological changes that are underpinned by increased neural processing which is required to interpret complex visual information for flight behavior. Anatomically, expansion of the mushroom body neurophil space in the brain and decrease in the volume of the olfactory glomeruli of the antennal lobes occurs during this transition [43]. Olfactory sensory neurons from the antennae project onto the glomeruli of the antennal lobe via the antennal nerve, and olfactory information is processed and projected to higher-order brain centers such as the mushroom bodies or lateral protocerebrum.

**Figure 6:**
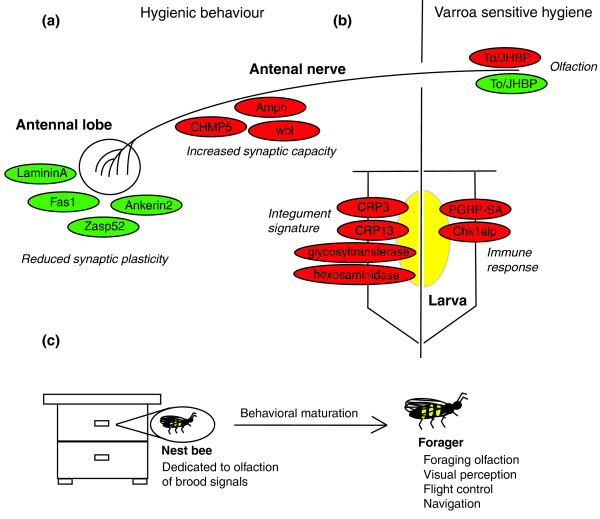
**Summary model for proteins identified in antennae and larvae from correlation screen**. For details see discussion.

The data presented here indicates that cells (most likely neurons with antennal axons) of bees performing rapid hygiene express different levels of proteins involved in adhesion and vesicle processing (Figure 6a), supporting the role of octopamine and maturation as an important control of this behavior. The cell adhesion proteins identified were all integrin proteins, some of which have been reported to regulate synaptic plasticity [44]. Specifically, ankerin 2 stabilizes synaptic connections to the spectrin-actin cytoskeleton and laminin A, Zasp and Fas1 are involved in the assembly of functional integrin adhesion sites essential for growth cone extension in axon guidance during neurogensis [45]. The increased expression of vesicle sorting proteins in hygienic bees indicates that while plasticity may be reduced, antennae of hygienic bees provide a strong input into higher brain function. These data could be explained by the environment of a hygienic nest bee, in which strong brood and queen-based olfactory cues are the major sensory inputs for bee development, behavior and social cohesion [43] (Figure 6d). Dimorphism in neural plasticity has been well characterized in the antennae of drones, where the antennal sensory nerves are thicker but project into a smaller number of glomeruli than in workers [46,47]. This configuration provides drones with the lower limit of detection for queen pheromone, enabling efficient queen finding during mating flights.

VSH limits mite reproductive success in the brood by specifically detecting the presence of a post-ovipositional mite. As part of the bees' response, a sensitive adult uncaps and re-caps the cell, effectively inhibiting mite reproduction [48]. The signal being sensed in this process remains unknown, although it peaks between three and five days after the cell is initially capped, leading to speculation that VSH adult bees respond to temporal fluxes in pathology mediated by oviposition, wounding related stress responses, infections, and olfactory cues [48]. Correlation between VSH scores and two proteins encoding divergent members of the To/JHBP super-family suggest they may be functionally linked to the behavior; To/JHBPs contain a conserved ligand binding domain with differing affinities to small lipophilic molecules such as JH and the N-terminal signal peptide indicates that they are probably secreted into the hemolymph where they act as soluble receptors for their ligands [49,50]. In the honey bee genome there are eight To/JHBP genes, located at two distinct loci, and we see one protein from each loci, one positively correlated with VSH and one negatively correlated (Figure 6b). Biologically, this separation in the genome suggests divergent functions and this is further supported by their differential regulation in our study. One of these proteins is completely uncharacterized but in *Phormia regina *the ortholog of the other To/JHBP is thought to be involved in chemosensation in antennal olfaction and taste [49] leading to the attractive hypothesis that it is playing a similar role in sensing brood.

That sensory and neuronal processes have a link to disease tolerant behavior may be expected but, intriguingly, a class of proteins involved in larval cuticle formation/structure also emerged as likely candidates. An arthropod's cuticle forms the primary physical barrier to the environment so while the cuticle plays an obvious role in pathogen defense, how it may contribute to social immunity mechanisms is less clear. Cuticular lipids differ between bees depending upon caste and attacks by *V. destructor *can alter the composition in adults and larvae [51,52]. The role of the cuticle in social immunity is supported by the data presented here, which indicates that several proteins involved in forming and maintaining the cuticle are significantly correlated with disease tolerance behaviors of nurse bees (Figure 6c).

## Conclusions

Our analysis of tissue proteomes from a large cohort of commercial honey bee colonies provides new clues to the evolution of biochemical components facilitating adaptation to disease. The control of behavior potentially represents the most complex paradigm in all living creatures so its study in natural, outbred systems is fraught with many difficulties, explaining the lack of coherent mechanisms describing these processes. Honey bees live in eusocial colonies and provide a scalable system for the study of developmental social biology and the divisions of labor it defines. Our results represent indications of molecular mechanisms underlying innate and social immunity behaviors in honey bees and build upon previous work demonstrating adaption involving neural remodeling and odorant recognition. A focused investigation of the processes identified here will provide an explanation of how host-pathogen interactions drive selection to generate disease tolerant colonies.

## Materials and methods

### Reagents

All chemicals used were of analytical grade or better and all solvents were of HPLC-grade or better; all were obtained from ThermoFisher-Scientific (St. Waltham, MA, USA).

### Honey bee-Varroa populations and physiology

We established 40 genetically heterogeneous honey bee colonies at a research apiary (Grand Forks, BC, Canada) in the spring of 2009 by shaking workers into a large cage and then portioning them back into single Langstroth box colonies with nine frames in each. Selected queens were then introduced into each new colony with initial populations of 1 kg of bees with relatively uniform *V. destructor *infestation rates, varying among colonies from 6.2% to 7.6% per 100 adult bees. Colonies were allowed to develop for six weeks to allow worker populations to turn over and be composed of the introduced queen's progeny, at which point we evaluated each for physiological *V. destructor *interactions and HB. HB was measured as the proportion of sealed brood cells uncapped (U) and removed of pupae (R) within 24 and 48 hours of freeze-killing defined patches brood with liquid nitrogen; PH and BI were estimated as described [53]. The proportion of uncapped cells referred to all cells uncapped by nurse bees including those where the pupae had been removed and those where the pupae was still present at the time of the observation. To estimate the ND *V. destructor *in each colony we counted the number of mites captured on screened bottom boards over four 24-hour collection periods spanning a period of 10 days. The ND estimates were normalized by colony size using the total weight of bees to determine the number of bees in each colony. VSH was estimated as the production of sexually viable female offspring as described [12].

### Analysis matrix

We used triplex dimethylation labeling and generated a D-optimal design matrix to group the samples in blocks of three and assigned a label to each sample as described [20]. A randomized incomplete block design similar to what we have used previously was chosen to minimize the standard error of the estimate of the colony effect on protein expression level [20].

### Sample collection and protein preparation

The antennae and larvae from colonies were sampled in triplicate. Ten pairs of antennae from nurse bees and three fifth instar larvae were removed *in situ *and frozen on dry ice. Larvae were further dissected to remove the digestive tracts and free-flowing hemolymph with stability maintained in PBS (50 mM K_2_HPO_4_, 150 mM NaCl, pH 7.4) containing complete, EDTA-free protease inhibitor cocktail (Roche, Mississauga, ON, Canada). Both samples were washed three times in PBS and prepared using essentially the same method. Both tissues were homogenized in 50 mM Tris-HCl, 150 mM NaCl, 1% NP-40, 20 mM dithiolthretitol in a Fast Prep bead mill with 2.8 mm ceramic beads (MP Biomedicals, Santa Ana, CA, USA) using 1 or 3 cycles of 20 s at 6.5 M/s with cooling for larvae or antennae, respectively. Tissue lysates were clarified at 5,000 relative centrifugal force (rcf) for 5 min at 4°C before ethanol/sodium acetate precipitation [54]. Proteolytic digestion of 20 μg (antennal) and 50 μg (larval) total protein was carried out as described [54]) and samples were labeled by reductive dimethylation using formaldehyde isotopologues [55] with slight modifications [20]. After labeling, each sample was pooled as required by the experimental design and each pool was separated into five fractions (antennae) using C_18_-SCX-C_18 _STAGE tips [56] or into six fractions (larvae) by isoelectric focusing with the OFFGEL system (Agilent Technologies, Santa Clara, CA, USA) [57].

### Proteome screen

Quantitative proteomic datasets were generated for antennae exactly as described in [20]. For larval tissue, LC-MS was done on a 1200 Series nanoflow HPLC system (Agilent Technologies) interfaced with a chromatin immunoprecipitation (CHIP)-cube to a 6520 Q-TOF (Agilent Technologies). Peptide separation was performed by reversed phase chromatography using a micro-fluidic CHIP comprised of an analytical column (75 μm ID, 150 mm length with a 300 Å C_18 _stationary phase) and a 160 nL trap column of the same phase. Peptides were loaded in 5% (v/v) acetonitrile, 0.1% (v/v), formic acid at 0.3 μL/min and then resolved at 0.3 μL/min for 90 min, during which a linear gradient of acetonitrile was created from 5% to 50% in 0.1% (v/v) formic acid. Mass spectrometry: Operating in auto MS/MS acquisition mode, the Q-TOF was set up to acquire full scan data over a mass range of 350 to 2,000 m/z and MS^2 ^for the six most intense, multiply-charged ions. Peak lists were created using Spectrum Mill extractor specifying fixed modification carboxyamidomethylation (C) and triplex dimethyl-mix (K,N-term) which accounts for all possible label moieties. Scans were merged within +/- 45 s elution time and maximum m/z window of 0.5 Da (usually 20 ppm). For database searching, oxidation (M) was added as a variable modification, peptide tolerance was +/- 20 ppm and fragment ion tolerance was 50 ppm, dynamic peak thresholding was switched on. Search results from Spectrum Mill were validated using autovalidation for protein score >20; (charge 2, Score >5, % SPI <50, Fwd - Rev Score >1, Rand 1-2 score >1), (charge 1, Score >6, % SPI <60, Fwd - Rev Score >1, Rand 1-2 score >1), (charge 3, Score >5, % SPI <50, Fwd - Rev Score >1, Rand 1-2 score >1) and for peptide hits (charge 2, Score >12, % SPI <60, Fwd - Rev Score >2, Rand 1-2 score >2), (charge 1, Score >8, % SPI <70, Fwd - Rev Score >2, Rand 1-2 score >2), (charge 2, Score >12, % SPI <60, Fwd - Rev Score >2, Rand 1-2 score >2). Both larvae and antennae datasets have peptide identification false discovery rates (FDR) well below 1% and were compiled into protein data arrays as described [20]. All experimental design and proteomic results are listed in Additional files 1, 2, 3 and 4.

### Data availability

All MS/MS data used in this study have been made available in two locations: they have all been deposited into the Honey Bee PeptideAtlas [58,59] as processed spectra and the raw files themselves are available on our FTP site (ftp://foster.chibi.ubc.ca/Downloads/BeeBiomarkers/).

### Statistical analysis

Logarithms of intensities were normalized by first subtracting the average of the three measurements in each block (for each protein independently) and then centering and standardizing within each label (across proteins) by the median and median absolute deviation [20]. For each protein, a linear mixed effects model was used to estimate the effect of each predictor variable on the protein expression level, adjusting for block and label factors. Colony was treated as a random factor to control for the three repeated measures within each colony. For each predictor variable an estimated effect, standard error and *P*-value was computed for each protein response. FDRs (q-values) were computed for the set of *P*-values of a given predictor over all protein response variables to adjust for multiple comparisons. All calculations were performed in the R statistical language.

### Gene enrichment analysis

Analysis was performed using Exploratory Gene Association Networks (EGAN) software [60] with pre collated networks for *Drosophila melanogaster *(Dmel). *A. mellifera *(Amel) gene identifiers were mapped to Dmel orthologs using the Round-Up database [61] and any unmapped Amel identifiers were assigned functions based on their closest homolog in *D. melanogaster *using BLAST-P, resulting in a total of 90% coverage for all antennal proteins identified. The remaining 10% were dealt with manually by drawing information from several sources: 1) honey bee genes othologs implicated in immunity [61]; 2) proteins found significantly regulated in response to bacterial infection by *Paenibacillus larvae *[63]; 3) proteins regulated in response to *V. destructor *infestation [18]; and 4) proteins specific to colony collapse disorder (CCD) affected colonies [64]. Enrichment analysis of proteins whose expression levels correlated (*P *< .05) with behavior and their direction of regulation was carried out by integrating these nodes with gene ontology nodes for component, function and process. For each node, over-representation or enrichment analysis was carried out employing a standard one-tailed Fisher's exact (hypergeometric) test using the entire gene list as background. Heat maps representing the interaction of important genes and their relationships by nodes were generated for both datasets independently.

## Abbreviations

Apo: apolipoprotein; BI: brood infestation; CCD: colony collapse disorder; CHIP: chromatin immunoprecipitation; Fas1: fasciclin 1; FDR: false discovery rate; Gasp: gene analogous to small peritrophins; HB: hygienic behavior; HPLC: high pressure liquid chromatography; JH: juvenile hormone; LC: liquid chromatography; LPS: lipopolysaccharide; LTA: lipoteichoic acid; MS: mass spectrometry; ND: natural drop; PBS: phosphate buffered saline; Pgm: phosphoglucomutase; PG: petidoglycan; PGRP-SA: peptidoglycan recognition protein SA; PH: phoretic; PPMC: Pearson's Product movement correlation; QTL: quantitative trait loci; RCF: relative centrifugal force; STAGE: stop and go extraction; To/JHBP: take-out/juvenile hormone binding protein; VSH: Varroa sensitive hygiene; Zasp: Z band alternately spliced PDZ-motif protein.

## Competing interests

The authors declare that they have no competing interests.

## Authors' contributions

APM, SFP, MMG, LH and LJF designed the experiments. LH maintained the bees and was assisted in performing behavioral assays by APM and SFP. LJF, KM, MMG and RP dissected the bees. RP and KM performed the biochemistry and mass spectrometry, as well as the functional bioinformatics. LJF authored the scripts for compiling proteomic data. RW designed and performed the statistical analyses. RP, LJF and MMG interpreted the results and wrote the initial version of the manuscript. All authors have read and approved the manuscript for publication.

## Supplementary Material

Additional file 1 **Spreadsheet of five pages for larval dataset**. Page 1, *P *values and protein annotation. For all proteins identified and quantitated the resultant *P *value from the correlation analysis with each trait and refseq annotation. Page 2, T statistic for each correlation provides a standardized value for the rate of change for each protein against per unit of each trait measured. Page 3, EGAN mapping, honey bee protein GI mapped to NCBI fly gene id. Page 4, *P *values for biological process gene enrichment for protein groups created by trait correlation and direction of correlation. Page 5, *P *values for global biological process gene enrichments. Click here for file

Additional file 2 **Spreadsheet of five pages for antenna dataset**. Page 1, *P *values and protein annotation. For each protein identified the resultant *P *value from the correlation analysis with each trait and refseq annotation is provided. Page 2, T statistic for each correlation provides a standardized value for the rate of change for each protein against per unit of each trait measured. Page 3, EGAN mapping, honey bee protein GI mapped to NCBI fly gene id. Page 4, *P *values for biological process gene enrichment for protein groups created by trait correlation and direction of correlation. Page 5, *P *values for global biological process gene enrichments. Click here for file

Additional file 3 **Spreadsheet of five pages for processed antenna LC-MS data**. Page 1 gives the experimental design designating used to generate LC-MS datasets. Each block is shown with colony numbers and the output file name generated for each block. Pages 2 to 4 are examples of the output from LC-MS and protein identification and quantitification result for each peptide used to generate proteins ratios. Page 5 is the result of rolling all peptide identification up into unique proteins using parsimony rules. Click here for file

Additional file 4 **Spreadsheet of five pages for processed larval LC-MS data**. Page 1 gives the experimental design designating used to generate LC-MS datasets. Each block is shown with colony numbers and the output file name generated for each block. Pages 2 to 4 are examples of the output from LC-MS and protein identification and quantitification result for each peptide used to generate proteins ratios. Page 5 is the result of rolling all peptide identification up into unique proteins using parsimony rules. Click here for file

## References

[B1] Evans JD Spivak M Socialized medicine: individual and communal disease barriers in honey bees J Invertebr Pathol 2010 103 S62 S72 19909975 10.1016/j.jip.2009.06.019

[B2] Anderson DL Trueman JWH Varroa jacobsoni (Acari: Varroidae) is more than one species Exp Appl Acarol 2000 24 165 189 10.1023/A:1006456720416 11108385

[B3] Le Conte Y Ellis M Ritter W Varroa mites and honey bee health: can Varroa explain part of the colony losses? Apidologie 2010 41 353 363 10.1051/apido/2010017

[B4] Rosenkranz P Aumeier P Ziegelmann B Biology and control of Varroa destructor J Invertebr Pathol 2010 103 S96 S119 19909970 10.1016/j.jip.2009.07.016

[B5] Evans JD Aronstein K Chen YP Hetru C Imler JL Jiang H Kanost M Thompson GJ Zou Z Hultmark D Immune pathways and defence mechanisms in honey bees Apis mellifera Insect Mol Biol 2006 15 645 656 10.1111/j.1365-2583.2006.00682.x 17069638 PMC1847501

[B6] Rothenbuhler WC Behavior genetics of nest cleaning in honey bees. IV. Responses of Fx and backcross generations to disease-killed brood Am Zool 1964 4 111 123 14172721 10.1093/icb/4.2.111

[B7] Harbo J Harris J Suppressed mite reproduction explained by the behaviour of adult bees J Apicultural Res 2005 44 21 23

[B8] Ibrahim A Spivak M The relationship between hygienic behavior and suppression of mite reproduction as honey bee (Apis mellifera) mechanisms of resistance to Varroa destructor Apidologie 2006 37 31 40 10.1051/apido:2005052

[B9] Spivak M Reuter GS Performance of hygienic honey bee colonies in a commercial apiary Apidologie 1998 29 291 302 10.1051/apido:19980308

[B10] Harbo JR Harris JW Responses to Varroa by honey bees with different levels of Varroa Sensitive Hygiene J Apicultural Res 2009 48 156 161 10.3896/IBRA.1.48.3.02

[B11] Spivak M Downey DL Field assays for hygienic behavior in honey bees (Hymenoptera: Apidae) J Econ Entomol 1998 91 64 70

[B12] Harbo JR Harris JW Resistance to Varroa destructor (Mesostigmata: Varroidae) when mite-resistant queen honey bees (Hymenoptera: Apidae) were free-mated with unselected drones J Econ Entomol 2001 94 1319 1323 10.1603/0022-0493-94.6.1319 11777031

[B13] Pernal S Sewalem A Melathopoulos A Breeding for hygienic behaviour in honeybees (Apis mellifera ) using free-mated nucleus colonies Apidologie 2012 43 403 424 10.1007/s13592-011-0105-x

[B14] Rinderer TE Harris JW Hunt GJ de Guzman LI Breeding for resistance to Varroa destructor in North America Apidologie 2010 41 409 424 10.1051/apido/2010015

[B15] Oxley PR Spivak M Oldroyd BP Six quantitative trait loci influence task thresholds for hygienic behaviour in honeybees (Apis mellifera) Mol Ecol 2010 19 1452 1461 10.1111/j.1365-294X.2010.04569.x 20298472

[B16] Gregory PG Evans JD Rinderer T de Guzman L Conditional immune-gene suppression of honeybees parasitized by Varroa mites J Insect Sci 2005 5 7 16299597 10.1093/jis/5.1.7 PMC1283888

[B17] Navajas M Migeon A Alaux C Martin-Magniette ML Robinson GE Evans JD Cros-Arteil S Crauser D Le Conte Y Differential gene expression of the honey bee Apis mellifera associated with Varroa destructor infection BMC Genomics 2008 9 301 10.1186/1471-2164-9-301 18578863 PMC2447852

[B18] Zhang Y Liu X Zhang W Han R Differential gene expression of the honey bees Apis mellifera and A. cerana induced by Varroa destructor infection J Insect Physiol 2010 56 1207 1218 10.1016/j.jinsphys.2010.03.019 20346951

[B19] Le Conte Y Alaux C Martin JF Harbo JR Harris JW Dantec C Severac D Cros-Arteil S Navajas M Social immunity in honeybees (Apis mellifera): transcriptome analysis of varroa-hygienic behaviour Insect Mol Biol 2011 20 399 408 10.1111/j.1365-2583.2011.01074.x 21435061

[B20] Parker R Melathopoulos AP White R Pernal SF Guarna MM Foster LJ Ecological adaptation of diverse honey bee (Apis mellifera) populations Plos One 2010 5 e11096 10.1371/journal.pone.0011096 20559562 PMC2886107

[B21] Lamant M Smih F Harmancey R Philip-Couderc P Pathak A Roncalli J Galinier M Collet X Massabuau P Senard JM Rouet P ApoO, a novel apolipoprotein, is an original glycoprotein up-regulated by diabetes in human heart J Biol Chem 2006 281 36289 36302 10.1074/jbc.M510861200 16956892

[B22] Hong S-T Bang S Hyun S Kang J Jeong K Paik D Chung J Kim J cAMP signalling in mushroom bodies modulates temperature preference behaviour in Drosophila Nature 2008 454 771 775 18594510 10.1038/nature07090

[B23] Yanay C Morpurgo N Linial M Evolution of insect proteomes: insights into synapse organization and synaptic vesicle life cycle Genome Biol 2008 9 R27 10.1186/gb-2008-9-2-r27 18257909 PMC2374702

[B24] Jung D-H Park H-J Byun H-E Park Y-M Kim T-W Kim B-O Um S-H Pyo S Diosgenin inhibits macrophage-derived inflammatory mediators through downregulation of CK2, JNK, NF-kappa B and AP-1 activation Int Immunopharmacol 2010 10 1047 1054 10.1016/j.intimp.2010.06.004 20601188

[B25] Bischoff V Vignal C Boneca IG Michel T Hoffmann JA Royet J Function of the drosophila pattern-recognition receptor PGRP-SD in the detection of Gram-positive bacteria Nat Immunol 2004 5 1175 1180 10.1038/ni1123 15448690

[B26] Michel T Reichhart JM Hoffmann JA Royet J Drosophila Toll is activated by Gram-positive bacteria through a circulating peptidoglycan recognition protein Nature 2001 414 756 759 10.1038/414756a 11742401

[B27] van der Horst DJ van Hoof D van Marrewijk WJ Rodenburg KW Alternative lipid mobilization: the insect shuttle system Mol Cell Biochem 2002 239 113 119 10.1023/A:1020541010547 12479576

[B28] Li WH Tanimura M Luo CC Datta S Chan L The apolipoprotein multigene family: biosynthesis, structure, structure-function relationships, and evolution J Lipid Res 1988 29 245 271 3288703

[B29] Whitten MM Tew IF Lee BL Ratcliffe NA A novel role for an insect apolipoprotein (apolipophorin III) in beta-1,3-glucan pattern recognition and cellular encapsulation reactions J Immunol 2004 172 2177 2185 14764684 10.4049/jimmunol.172.4.2177

[B30] Garedew A Schmolz E Lamprecht I The energy and nutritional demand of the parasitic life of the mite Varroa destructor Apidologie 2004 35 419 430 10.1051/apido:2004032

[B31] de D'Aubeterre JP Myrold DD Royce LA Rossignol PA A scientific note of an application of isotope ratio mass spectrometry to feeding by the mite, Varroa jacobsoni Oudemans, on the honeybee, Apis mellifera L Apidologie 1999 30 351 352 10.1051/apido:19990413

[B32] Tewarson NC Singh A Engels W Reproduction of Varroa-Jacobsoni in colonies of Apis cerana-indica under natural and experimental conditions Apidologie 1992 23 161 171 10.1051/apido:19920209

[B33] Feldhaar H Gross R Immune reactions of insects on bacterial pathogens and mutualists Microbes Infect 2008 10 1082 1088 10.1016/j.micinf.2008.07.010 18672091

[B34] Dushay MS Insect hemolymph clotting Cell Mol Life Sci 2009 66 2643 2650 10.1007/s00018-009-0036-0 19418022 PMC11115950

[B35] Kanbar G Engels W Ultrastructure and bacterial infection of wounds in honey bee (Apis mellifera) pupae punctured by Varroa mites Parasitol Res 2003 90 349 354 10.1007/s00436-003-0827-4 12684884

[B36] Jensen K de Miranda Santos IK Glass EJ Using genomic approaches to unravel livestock (host)-tick-pathogen interactions Trends Parasitol 2007 23 439 444 10.1016/j.pt.2007.07.006 17656152

[B37] Yang X Cox-Foster DL Impact of an ectoparasite on the immunity and pathology of an invertebrate: evidence for host immunosuppression and viral amplification Proc Natl Acad Sci USA 2005 102 7470 7475 10.1073/pnas.0501860102 15897457 PMC1140434

[B38] Richards EH Jones B Bowman A Salivary secretions from the honeybee mite, Varroa destructor: effects on insect haemocytes and preliminary biochemical characterization Parasitology 2011 138 602 608 10.1017/S0031182011000072 21281563

[B39] Navajas M Migeon A Alaux C Martin-Magniette M Robinson G Evans J Cros-Arteil S Crauser D Le Conte Y Differential gene expression of the honey bee Apis mellifera associated with Varroa destructor infection BMC Genomics 2008 9 301 10.1186/1471-2164-9-301 18578863 PMC2447852

[B40] Swanson JAI Torto B Kells SA Mesce KA Tumlinson JH Spivak M Odorants that induce hygienic behavior in honeybees: identification of volatile compounds in chalkbrood-infected honeybee larvae J Chem Ecol 2009 35 1108 1116 10.1007/s10886-009-9683-8 19816752

[B41] Spivak M Masterman R Ross R Mesce KA Hygienic behavior in the honey bee (Apis mellifera L.) and the modulatory role of octopamine J Neurobiol 2003 55 341 354 10.1002/neu.10219 12717703

[B42] Schulz DJ Barron AB Robinson GE A role for octopamine in honey bee division of labor Brain Behav Evol 2002 60 350 359 10.1159/000067788 12563167

[B43] Fahrbach SE Robinson GE Juvenile hormone, behavioral maturation, and brain structure in the honey bee Dev Neurosci 1996 18 102 114 10.1159/000111474 8840089

[B44] Koch I Schwarz H Beuchle D Goellner B Langegger M Aberle H Drosophila ankyrin 2 is required for synaptic stability Neuron 2008 58 210 222 10.1016/j.neuron.2008.03.019 18439406

[B45] Dent EW Gupton SL Gertler FB The growth cone cytoskeleton in axon outgrowth and guidance Cold Spring Harb Perspect Biol 2011 doi: 10.1101/cshperspect.a001800 10.1101/cshperspect.a001800 PMC3039926 21106647

[B46] Arnold G Masson C Budharugsa S Comparative study of the antennal lobes and their afferent pathway in the worker bee and the drone (Apis mellifera) Cell Tissue Res 1985 242 593 605

[B47] Brockmann A Bruckner D Structural differences in the drone olfactory system of two phylogenetically distant Apis species, A-florea and A-mellifera Naturwissenschaften 2001 88 78 81 10.1007/s001140000199 11320892

[B48] Harris JW Bees with Varroa Sensitive Hygiene preferentially remove mite infested pupae aged <= five days post capping J Apicultural Res 2007 46 134 139

[B49] Fujikawa K Seno K Ozaki M A novel Takeout-like protein expressed in the taste and olfactory organs of the blowfly, Phormia regina FEBS J 2006 273 4311 4321 10.1111/j.1742-4658.2006.05422.x 16930135

[B50] Hagai T Cohen M Bloch G Genes encoding putative Takeout/juvenile hormone binding proteins in the honeybee (Apis mellifera) and modulation by age and juvenile hormone of the takeout-like gene GB19811 Insect Biochem Mol Biol 2007 37 689 701 10.1016/j.ibmb.2007.04.002 17550825

[B51] Del Piccolo F Nazzi F Della Vedova G Milani N Selection of Apis mellifera workers by the parasitic mite Varroa destructor using host cuticular hydrocarbons Parasitology 2010 137 967 973 10.1017/S0031182009991867 20152062

[B52] Salvy M Martin C Bagneres AG Provost E Roux M Le Conte Y Clement JL Modifications of the cuticular hydrocarbon profile of Apis mellifera worker bees in the presence of the ectoparasitic mite Varroa jacobsoni in brood cells Parasitology 2001 122 145 159 11272645 10.1017/s0031182001007181

[B53] Harris JW Harbo JR Villa JD Danka RG Variable population growth of Varroa destructor (Mesostigmata: Varroidae) in colonies of honey bees (Hymenoptera: Apidae) during a 10-year period Environ Entomol 2003 32 1305 1312 10.1603/0046-225X-32.6.1305

[B54] Foster LJ de Hoog CL Mann M Unbiased quantitative proteomics of lipid rafts reveals high specificity for signaling factors Proc Natl Acad Sci USA 2003 100 5813 5818 10.1073/pnas.0631608100 12724530 PMC156283

[B55] Boersema PJ Aye TT van Veen TAB Heck AJR Mohammed S Triplex protein quantification based on stable isotope labeling by peptide dimethylation applied to cell and tissue lysates Proteomics 2008 8 4624 4632 10.1002/pmic.200800297 18850632

[B56] Ishihama Y Rappsilber J Mann M Modular stop and go extraction tips with stacked disks for parallel and multidimensional peptide fractionation in proteomics J Proteome Res 2006 5 988 994 10.1021/pr050385q 16602707

[B57] Rogers LD Fang Y Foster LJ An integrated global strategy for cell lysis, fractionation, enrichment and mass spectrometric analysis of phosphorylated peptides Mol Biosyst 2010 6 822 829 10.1039/b915986j 20567768

[B58] Peptide Atlas http://www.peptideatlas.org/builds/honeybee/index.php

[B59] Chan QWT Parker P Sun Z Deutsch EW Foster LJ A honey bee (Apis mellifera L.) PeptideAtlas crossing castes and tissues BMC Genomics 2011 12 290 10.1186/1471-2164-12-290 21639908 PMC3213019

[B60] Paquette J Tokuyasu T EGAN: exploratory gene association networks Bioinformatics 2010 26 285 286 10.1093/bioinformatics/btp656 19933825 PMC2804305

[B61] DeLuca TF Wu IH Pu J Monaghan T Peshkin L Singh S Wall DP Roundup: a multi-genome repository of orthologs and evolutionary distances Bioinformatics 2006 22 2044 2046 10.1093/bioinformatics/btl286 16777906

[B62] Rannikko K Ortutay C Vihinen M Immunity genes and their orthologs: a multi-species database Int Immunol 2007 19 1361 1370 10.1093/intimm/dxm109 17965450

[B63] Chan QWT Melathopoulos AP Pernal SF Foster LJ The innate immune and systemic response in honey bees to a bacterial pathogen, Paenibacillus larvae BMC Genomics 2009 10 387 10.1186/1471-2164-10-387 19695106 PMC2907699

[B64] Johnson RM Evans JD Robinson GE Berenbaum MR Changes in transcript abundance relating to colony collapse disorder in honey bees (Apis mellifera) Proc Natl Acad Sci USA 2009 106 14790 14795 10.1073/pnas.0906970106 19706391 PMC2736458

